# Supplementary figures and images for: Water tank and swimming pool detection based on remote sensing and deep learning: Relationship with socioeconomic level and applications in dengue control
Source: PLoS One. 2021 Dec 9;16(12):e0258681. doi: 10.1371/journal.pone.0258681 (PMC8659416; doi:10.1371/journal.pone.0258681)

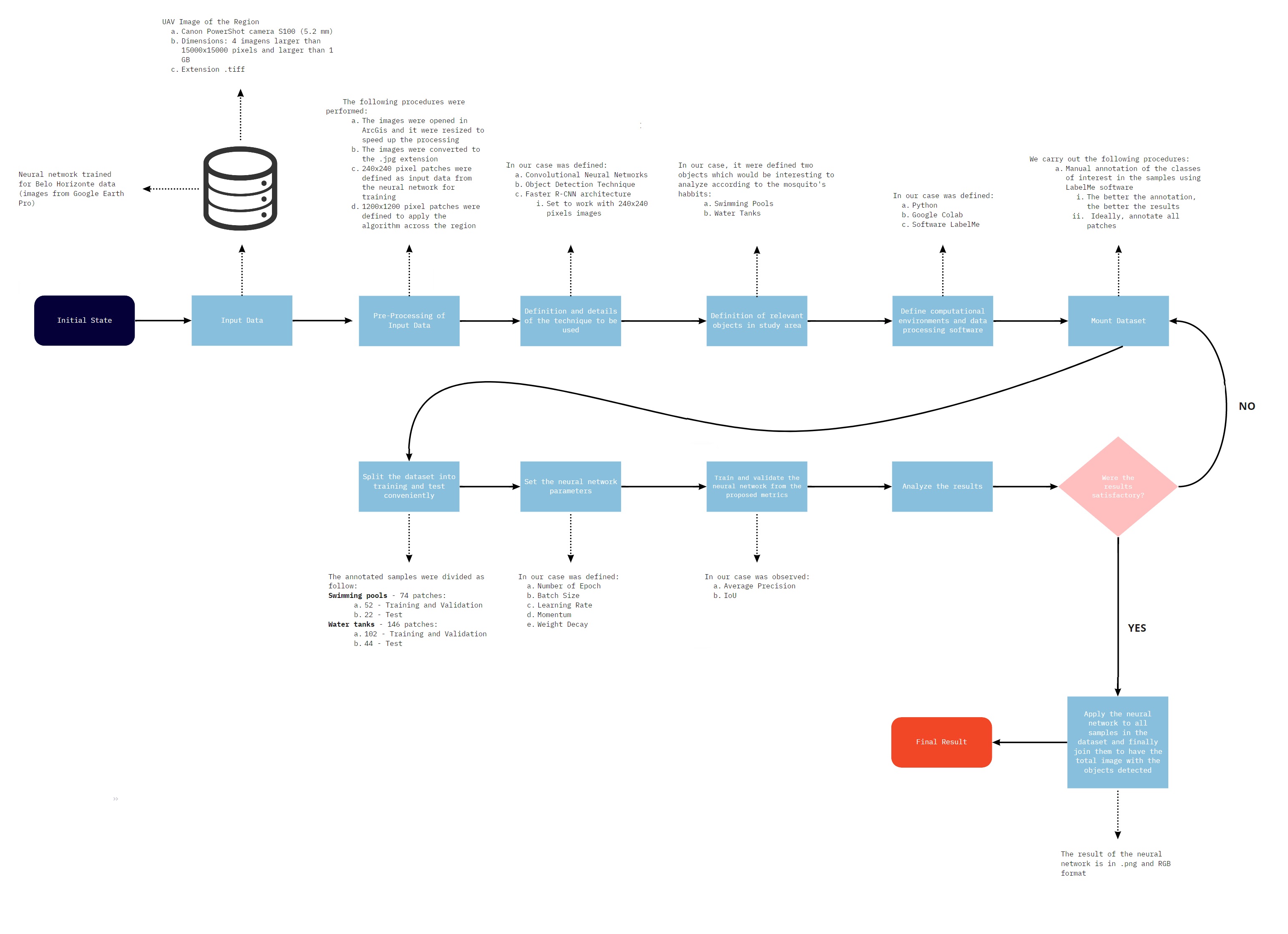

Supplement: S1 Fig — The flowchart illustrates the process performed to create the neural network models responsible for water tanks and swimming pools detection in digital images. (JPG) [file pone.0258681.s001.jpg]

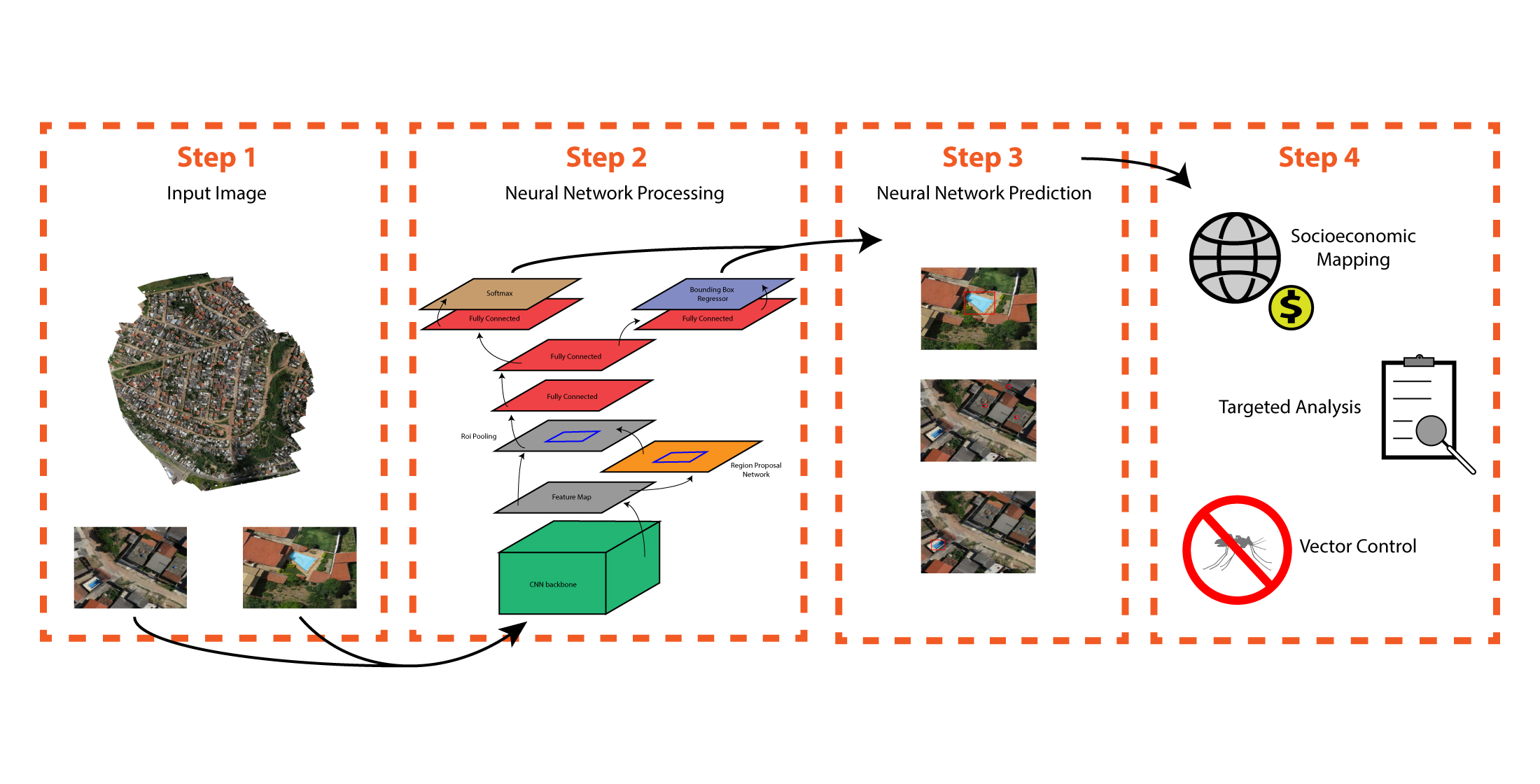

Supplement: S2 Fig — This image illustrates the steps that guide the study performed. (JPG) [file pone.0258681.s002.jpg]
